# GenAI-mediated immersive dialogue practice in liaison interpreting: a mixed-methods investigation of cognitive-affective outcomes and interactional processes

**DOI:** 10.3389/fpsyg.2026.1914690

**Published:** 2026-09-29

**Authors:** Hui Zhang, Paramasivam Muthusamy, Gang Qin

**Affiliations:** 1 Bonong College of Agricultural Science and Technology, Zhoukou Normal University, Zhoukou, China; 2 Faculty of Education and Liberal Arts, INTI International University, Putra Nilai, Malaysia; 3 School of Artificial Intelligence, Zhoukou Normal University, Zhoukou, China

**Keywords:** cognitive load, generative AI, immersive dialogue practice, interpreting anxiety, liaison interpreting

## Abstract

**Introduction:**

Liaison interpreting competence encompasses working memory management, executive control, self-efficacy, and affect regulation, yet its development remains constrained by the linguistic inconsistency and variable cognitive-affective demands of conventional peer role-play. Drawing on an integrated framework, this quasi-experimental mixed-methods study investigates whether Generative AI (GenAI)-mediated immersive dialogue practice can create a more cognitively authentic and affectively facilitative practice environment for interpreter-learners.

**Method:**

Seventy undergraduate students from two intact classes in a Chinese university were assigned to an experimental group (*n* = 35) practicing with a voice-enabled GenAI tool simulating bilingual interlocutor personas, or a control group (*n* = 35) engaged in traditional peer role-play over 8 weeks. Because only one class was assigned to each condition, the between-group comparisons are exploratory. Data included interpreting performance scores, anxiety and engagement questionnaires, coded interaction transcripts analyzed through Epistemic Network Analysis (ENA), and semi-structured interviews examined via reflexive thematic analysis.

**Results and discussion:**

The experimental group scored significantly higher than the control group in overall interpreting performance, particularly in informational accuracy and interactional management. GenAI-supported practice was also associated with significantly lower interpreting-related anxiety with a large effect size. Learning engagement did not differ significantly, indicating that the two modalities may sustain engagement through different psychological mechanisms. Interaction analysis revealed a hub-and-spoke network centered on meaning transfer, with clarification requests and self-repair reflecting active metacognitive monitoring. This pattern represents the aggregate structural profile of interaction across the intervention period rather than a developmental trajectory. Qualitative findings identified psychological safety and repeatable mastery opportunities as key affordances, alongside role instability and limited corrective feedback as constraints, with students exercising psychological agency through prompt negotiation and self-directed scaffolding. These findings offer preliminary empirical support for GenAI as an affectively facilitative practice partner whose effects are theoretically consistent with predictions derived from Cognitive Load Theory, Processing Efficiency Theory, and Self-Efficacy Theory, although the underlying cognitive and motivational mechanisms remain to be directly tested.

## 1 Introduction

### 1.1 Cognitive-affective demands of liaison interpreting and the challenge of authentic practice

Liaison interpreting is a cognitively and affectively demanding communicative activity that differs from other interpreting modes in its immediacy, face-to-face nature, and fundamentally dialogic character (Wadensjö, 2014; Angelelli, 2004). Unlike simultaneous interpreting, which often positions the interpreter outside the direct social exchange, liaison interpreting requires the interpreter to function as a visible participant in a communicative triad, managing cross-linguistic transfer, interactional coordination, and socio-pragmatic monitoring simultaneously under real-time pressure (Pöchhacker, 2022; Hale, 2007; Gile, 2009). This constellation of demands—spanning working memory, executive control, affect regulation, and self-efficacy maintenance—means that the development of liaison interpreting competence depends heavily on sustained exposure to authentic, cognitively challenging, and affectively manageable interactional situations (Bontempo and Napier, 2011; Bandura, 1997).

Despite this widely acknowledged need, liaison interpreting training still relies predominantly on peer role-play (Gile, 2009; Setton and Dawrant, 2016). While peer role-play offers valuable collaborative learning opportunities, it presents recurring limitations: student peers often lack the domain-specific terminology, stable institutional register, and sustained role coherence needed to portray professional stakeholders convincingly (Sandrelli and de Manuel Jerez, 2007). As a result, the cognitive and affective demands generated during practice can vary substantially across student pairs and often fail to approximate those of authentic professional encounters, making practice quality inconsistent and difficult for instructors to calibrate (Qu and Chen, 2026; Braun et al., 2020). Technology-enhanced alternatives such as pre-recorded video scenarios have partially improved input quality but cannot reproduce the dynamic, contingent turn-taking central to liaison interpreting (Gieshoff et al., 2025). These limitations point to a clear gap between the psychological demands of professional liaison interpreting and the capacity of existing practice tools to engage those demands authentically.

To address this gap, the present study proposes the integration of GenAI-mediated immersive dialogue practice—using adaptive, persona-driven dialogue generation to expose interpreter-learners to terminologically dense, register-consistent, and context-specific communicative input (Miner and Nicodemus, 2021; Qu and Chen, 2026). The study adopts an integrated cognitive-affective-motivational framework (detailed in Section 2.2) to examine how this technology-mediated practice environment shapes interpreting competence development in terms of both cognitive-affective outcomes and the interactional processes through which learning unfolds.

### 1.2 Technological affordances of GenAI and research gaps

Recent advances in voice-enabled Generative Artificial Intelligence (GenAI), particularly those powered by Multimodal Large Language Models (MLLMs), offer important technological possibilities for creating such psychologically grounded immersive practice environments (Goh and Aryadoust, 2025; Lee et al., 2026; Wang et al., 2024). Unlike earlier rule-based conversational agents that operated within narrowly predefined scripts, contemporary large language model-based systems can adopt highly specific personas, generate context-sensitive responses, and maintain consistent domain-specific registers and discourse strategies over extended interactions (Kasneci et al., 2023; Kohnke et al., 2023). Through carefully designed prompts, GenAI can function as a responsive and versatile interlocutor (Jindapitak and Tantiwich, 2025; Qu and Chen, 2026), creating a simulated yet communicatively rich environment in which students negotiate meaning across languages, manage turn-taking, address communication breakdowns, and rehearse interpreting strategies. From a cognitive-psychological standpoint, such environments can be calibrated to provide optimal cognitive challenge—sufficiently complex to promote schema acquisition and working memory development, yet sufficiently structured to prevent cognitive overload (Sweller et al., 2011). From an affective perspective, the non-evaluative and repeatable nature of GenAI interaction may reduce social-evaluative anxiety (Horwitz et al., 1986), creating conditions that foster willingness to take communicative risks and thereby promote self-efficacy development (Bandura, 1997; Gnewuch and Reinkemeier, 2026; Lukešová and Jennings, 2025).

Despite growing scholarly interest in GenAI applications in language-related fields, empirical research on its psychological effects in liaison interpreting practice remains limited. Existing studies have focused mainly on GenAI as a tool for machine translation evaluation or automated written feedback (e.g., Kornacki and Pietrzak, 2024; Guo and Wang, 2024), with particular attention to product-oriented outcomes such as translation accuracy (Jiao et al., 2023). By contrast, process-oriented research examining the cognitive and affective dynamics of how interpreting students interact with GenAI in real-time dialogic settings remains scarce. In particular, little is known about the cognitive strategies learners deploy, the self-regulatory mechanisms they activate, and the interactional patterns that emerge during GenAI-mediated interpreting practice. The affective dimensions of this form of practice, including its impact on self-efficacy beliefs, engagement, and interpreting-related anxiety, have also received limited empirical attention. Moreover, as GenAI shifts from a passive support tool to an interactive conversational partner, it becomes increasingly important to understand how learners exercise psychological agency—making autonomous decisions about strategy use, help-seeking, and communicative risk-taking—within these technology-mediated practice environments (Lantolf and Beckett, 2009; Bandura, 2006). A systematic investigation of the cognitive, affective, and agentive dynamics of GenAI-mediated interpreting practice is therefore needed to advance both theoretical understanding and practice design in this emerging area.

To address these gaps, the present study examines the effectiveness of GenAI-mediated immersive dialogue practice in a university-level liaison interpreting course. Using a mixed-methods design that combines quantitative evidence with qualitative interaction analysis, the study seeks to capture both the outcomes and the processes of GenAI-supported interpreting practice. Specifically, the study is guided by the following research questions:

- RQ1: To what extent does GenAI-based role-play affect students’ liaison interpreting performance compared with traditional peer role-play?
- RQ2: To what extent is GenAI-based role-play associated with differences in students’ learning engagement and interpreting-related anxiety compared with traditional peer role-play?
- RQ3: What interaction patterns and meaning-negotiation strategies *characterize* students’ GenAI-based role-play?
- RQ4: How do students perceive the affordances and limitations of GenAI as an interactive partner, and how do they exercise agency within this GenAI-mediated learning environment?

The present study makes four contributions that distinguish it from existing work. Structurally, it operationalizes a human-AI communicative triad, in which one GenAI system sustains two bilingual interlocutor personas while the learner serves as the sole human mediator, a configuration unaddressed by either product-oriented GenAI interpreting research (Jia and Aryadoust, 2024; Lu, 2025; Zou et al., 2026) or dyadic AI-supported speaking studies (Chen et al., 2025; Wu and Liu, 2025). Theoretically, it advances an integrated cognitive–affective–motivational framework and translates it into three designable properties of the practice environment. Methodologically, it introduces a coding scheme that extends Wadensjö’s (2014) rendition/non-rendition distinction by adding a third dimension for human–AI collaboration and learner agency, analyzed through Epistemic Network Analysis. Empirically, it yields a differentiated account in which the divergence between learners’ reported and enacted agency converts a general endorsement of GenAI into a specific pedagogical requirement: agency must be scaffolded, not assumed.

## 2 Literature review

### 2.1 Cognitive, affective, and interactional demands of liaison interpreting

Liaison interpreting occupies a distinctive place within interpreting studies because it confronts interpreters with a unique constellation of cognitive, affective, and socio-interactional demands. Unlike conference interpreting, in which interpreters are often spatially removed from the primary speakers, liaison interpreting situates the interpreter as a visible participant in a triadic exchange (Wadensjö, 2014; Pöchhacker, 2022). From a cognitive-psychological perspective, this mode of interpreting constitutes an inherently dual-task activity. Gile's (2009) Effort Models characterize interpreting as a process in which listening, memory, production, and coordination efforts compete for limited processing capacity. In liaison interpreting, these demands are compounded by the need for real-time interactional management: the interpreter must not only relay utterances across languages but also coordinate turn-taking, request clarification, and regulate the flow of interaction (Wadensjö, 2014), all of which require executive control processes including inhibition, cognitive flexibility, and self-monitoring (Miyake et al., 2000; Diamond, 2013). When total processing demands exceed available working memory capacity, performance deteriorates (Timarová, 2008; Seeber, 2011). Angelelli (2004) further demonstrates that interpreters act as social agents who navigate power asymmetries and mediate cultural meanings, while Hale (2007) documents the pragmatic demands of register management and the conveyance of illocutionary force—capacities that require not only linguistic competence but also perspective-taking and socio-pragmatic sensitivity.

Beyond these cognitive demands, liaison interpreting imposes significant affective challenges. Interpreting-related anxiety, a situation-specific form of performance anxiety (Horwitz et al., 1986; Spielberger, 1966), has been identified as a pervasive concern among student interpreters (Chiang, 2010; Bontempo and Napier, 2011). According to Eysenck and Calvo's (1992) processing efficiency theory, anxiety generates task-irrelevant processing (worry) that competes with task-relevant processing for working memory resources, leading to performance decrements particularly under conditions of high cognitive load. Self-efficacy—the individual’s belief in their capacity to perform effectively (Bandura, 1997)—moderates this relationship: higher self-efficacy is associated with more adaptive appraisals of challenge and better preservation of cognitive resources under pressure (Bontempo and Napier, 2011). In addition, interpreters must engage in emotional regulation when managing distressing content or interpersonal tension, placing further demands on executive control (Gross, 1998; Korpal, 2016; Kurz, 2003). Taken together, this body of scholarship establishes that liaison interpreting competence is a multidimensional psychological construct encompassing working memory management, executive control, socio-pragmatic sensitivity, affect regulation, and self-efficacy, rather than bilingual proficiency alone.

Within interpreter education, peer-based role-play has long been the dominant approach for cultivating these competencies (Gile, 2009; Setton and Dawrant, 2016; González Davies, 2004). However, it presents recurring limitations when evaluated against the cognitive-affective demands outlined above. Student peers often lack the domain-specific lexical repertoire, institutional register, and sustained role coherence needed to represent professional stakeholders convincingly (Sandrelli and de Manuel Jerez, 2007). From the perspective of Cognitive Load Theory (Sweller et al., 2011), when source discourse is linguistically impoverished or register-inconsistent, the intrinsic cognitive load generated during practice fails to approximate that of authentic professional encounters, undermining the development of robust interpreting schemas (Qu and Chen, 2026; González-Davies and Raído, 2018). The inconsistency of peer performance also affects affective outcomes: unpredictable variation in task difficulty can undermine learners’ sense of competence and control, thereby impeding self-efficacy development (Bandura, 1997). In addition, classroom time constraints and a fixed pool of classmates restrict the range and volume of practice opportunities (Braun et al., 2020). Technology-enhanced alternatives such as pre-recorded video scenarios have partially improved input quality but cannot reproduce the dynamic turn-taking central to liaison interpreting (Gieshoff et al., 2025; Sandrelli and de Manuel Jerez, 2007). This gap between the complex cognitive-affective demands of liaison interpreting and the limited capacity of existing practice tools to engage these demands authentically motivates the present exploration of GenAI-mediated alternatives.

### 2.2 Theoretical framework: an integrated cognitive-affective-motivational perspective

The theoretical framework guiding this study integrates insights from four complementary psychological perspectives. Cognitive Load Theory (CLT) (Sweller, 1988; Sweller et al., 2011) posits that learning is constrained by limited working memory capacity and that effective practice environments should optimize the allocation of cognitive resources by generating sufficient intrinsic load (the complexity inherent in the material) to promote schema acquisition, while minimizing extraneous load (inefficiencies arising from inconsistent or poorly calibrated task design) and maximizing germane load (effort invested in building automated cognitive structures) (van Merriënboer and Sweller, 2005). Self-Efficacy Theory (Bandura, 1997) complements this cognitive account by explaining how practice experiences shape learners’ beliefs about their own capacity. Self-efficacy is most powerfully built through enactive mastery—direct performance in challenging but manageable tasks—and operates through cognitive, motivational, and affective channels, influencing both processing efficiency and persistence in the face of difficulty. Research on foreign language and performance anxiety (Horwitz et al., 1986; Eysenck and Calvo, 1992; MacIntyre and Gardner, 1994) adds a further dimension: anxiety consumes working memory through worry-related processing, and its effects are most pronounced under high cognitive load. Practice environments that reduce social-evaluative threat are therefore predicted to free working memory resources for task-relevant processing. Finally, Self-Determination Theory (SDT; Deci and Ryan, 2000; Ryan and Deci, 2017) addresses the motivational conditions for sustained engagement, positing that intrinsic motivation is supported when learners experience autonomy (volitional control over practice), competence (appropriate challenge and informative feedback), and relatedness (responsive interaction). While these four perspectives address intrapersonal psychological dynamics, the situated cognition perspective (Brown et al., 1989; Barsalou, 2020; Shapiro and Stolz, 2019) adds an ecological dimension: cognitive schemas, self-regulatory strategies, and self-efficacy beliefs developed in practice contexts that closely resemble professional settings are more likely to transfer to real-world performance (Kiraly, 2014; González-Davies and Raído, 2018; Calvo, 2015).

Building on these theoretical premises, the present study conceptualizes GenAI-mediated immersive dialogue practice as a psychologically grounded practice environment that operationalizes the integrated framework through three dimensions. The first is *cognitive authenticity*: the practice environment generates intrinsic cognitive load profiles approximating professional interpreting through terminological density, register consistency, and discourse complexity, which is predicted to support schema development (Sweller et al., 2011; Brown et al., 1989). The second is *affective optimization*: by reducing social-evaluative threat and providing repeatable mastery opportunities, the environment is hypothesized to promote self-efficacy development and reduce debilitative anxiety, potentially freeing cognitive resources for task-relevant processing (Bandura, 1997; Eysenck and Calvo, 1992). The third is *motivational scaffolding*: the environment supports autonomy (self-paced, self-directed practice), competence (appropriately challenging interaction), and a degree of relatedness (responsive conversational engagement), fostering the intrinsic motivation that SDT predicts will sustain effortful practice (Deci and Ryan, 2000). Viewed through this integrated lens, GenAI is not merely a passive support tool but a mechanism for constructing a practice environment that simultaneously engages the cognitive, affective, and motivational processes underlying interpreting competence development. The present study tests the predicted outcomes of this framework—interpreting performance, anxiety, and engagement—without directly measuring the underlying cognitive and motivational mechanisms (cognitive load, schema acquisition, self-efficacy). The theoretical interpretations offered in the Discussion should therefore be read as framework-consistent accounts rather than empirically verified pathways.

### 2.3 Generative AI in language and interpreting research

The emergence of generative artificial intelligence (GenAI), particularly large language models (LLMs) such as ChatGPT and Gemini, has significantly transformed the landscape of technology-mediated language practice. From a technical standpoint, GenAI is able to generate anthropomorphic, context-sensitive, and linguistically sophisticated discourse, thereby moving beyond the scripted limitations of earlier chatbot technologies (Law, 2024; Goh and Aryadoust, 2025). Recent scoping reviews suggest that its principal affordances include personalized feedback, content generation, and, most importantly for the present study, the simulation of interactive speaking partners (Lee et al., 2026; Lin et al., 2025). In English as a Foreign Language (EFL) settings, emerging empirical evidence indicates that GenAI-supported speaking practice can enhance learners’ oral proficiency and willingness to take linguistic risks while alleviating foreign language speaking anxiety (Chen et al., 2025; Wu and Liu, 2025). Fathi et al. (2024) demonstrated that AI-mediated speaking tasks significantly improved EFL learners’ speaking skills and willingness to communicate, while Tang et al. (2026) showed that self-efficacy and speaking enjoyment were key drivers of learners’ willingness to communicate with GenAI. These findings can be interpreted through the theoretical framework articulated above: GenAI may function as a psychologically facilitative practice partner because it reduces social-evaluative threat (lowering anxiety and freeing working memory resources), provides consistent and repeatable interaction (supporting self-efficacy through mastery experiences), and allows learner-controlled pacing and topic selection (supporting autonomy). Goh and Aryadoust (2025) characterize this as the co-intelligent nature of GenAI, which functions not simply as a tool but as an interactive interlocutor capable of sustaining extended, meaning-oriented exchanges over time.

Although the use of GenAI in language-related research has developed rapidly, its integration into interpreting practice and the investigation of its psychological effects on interpreter-learners remain comparatively limited. To date, most studies have focused on its value for discrete sub-skills or assessment-related tasks. Jia and Aryadoust (2024), for example, found that ChatGPT-4 demonstrated promising reliability in assessing interpreting accuracy. More recently, Han et al. (2025) conducted three inter-linked studies examining how LLMs perform in classroom-based assessment of English-Chinese consecutive interpreting, demonstrating that ChatGPT-generated feedback can rival teacher feedback in formative assessment contexts. Wang and Wang (2025) further investigated GPT and Claude for automatic assessment of target-language quality in interpreting, finding general alignment between AI-derived and human scores while identifying discrepancies in register, sentence complexity, and flow. In a quasi-experimental design, Yu et al. (2025) compared interpreting students’ engagement strategies with feedback from a GenAI chatbot and from peers, finding that students interacting with the chatbot reported significantly lower agreement and higher dispute with evaluative comments, suggesting that AI feedback may promote more critical engagement with performance assessment. Zou et al. (2026) similarly reported that GenAI could effectively scaffold students’ gisting skills, that is, their ability to extract core meaning under time pressure—a capacity closely related to working memory efficiency and attentional allocation (Timarová, 2008). Lu (2025) examined the effectiveness of ChatGPT in promoting learners’ lexical and syntactic flexibility during consecutive interpreting practice. In terms of learner perceptions, Fan et al. (2026) observed that although students remain cautious about the adoption of GenAI, they increasingly recognize its potential to provide immediate, personalized, and domain-specific feedback.

Despite these encouraging developments, an important gap remains between the technological affordances of GenAI and the understanding of its psychological effects on interpreter-learners during dialogic practice. Liaison interpreting is defined by its triadic, dialogic, and socio-interactional character, requiring interpreters to manage shifting cognitive demands, affective pressures, and interpersonal dynamics in real time (Angelelli, 2004; Wadensjö, 2014). As discussed in Section 2.1, these demands encompass working memory management, executive control, self-efficacy regulation, and anxiety management—psychological processes that are intimately shaped by the characteristics of the practice environment. In principle, contemporary LLMs possess the technical capacity to create practice environments that engage these processes by placing learners in persona-driven, register-consistent, complexity-adjustable, and unpredictable communicative settings. However, prior research has largely adopted a product-oriented perspective, emphasizing measurable outcomes such as translation accuracy or gisting performance (Jiao et al., 2023; Lu, 2025). Process-oriented research examining the cognitive strategies, self-regulatory mechanisms, and affective dynamics that unfold during GenAI-mediated interpreting practice remains scarce. In particular, little is known about how the practice environment shapes learners’ cognitive load management, self-efficacy development, and anxiety regulation in real time. Furthermore, the question of psychological agency—how learners make autonomous decisions about strategy use, help-seeking, and communicative risk-taking within AI-mediated practice (Bandura, 2006)—has received minimal empirical attention in interpreting contexts.

To address these gaps, the present study investigates the effectiveness of GenAI-mediated immersive dialogue practice in a university-level liaison interpreting course. Adopting a mixed-methods design, it examines both the outcomes of GenAI-supported practice—including interpreting performance, engagement, and anxiety—and the processes through which such practice unfolds, particularly the cognitive-affective-interactional dynamics that emerge during human-AI engagement. In doing so, the study aims to contribute an empirical and theoretical basis for understanding the psychological mechanisms through which technology-mediated practice environments support interpreting competence development, and to inform the design of psychologically grounded, interactive, and ecologically valid interpreting curricula in the age of artificial intelligence.

## 3 Method

This study employed an explanatory sequential mixed-methods design (Creswell and Plano Clark, 2018) in which quantitative data (interpreting performance, anxiety, engagement) addressed RQ1 and RQ2, coded interaction transcripts analyzed through frequency counts and Epistemic Network Analysis addressed RQ3, and qualitative interview data addressed RQ4. The four strands were integrated at the interpretation stage: the interview findings were used to contextualize and extend the quantitative and interaction-analytic results, providing participant-level explanations for patterns observed in the aggregate data. Only EG students were interviewed because RQ3 and RQ4 focused specifically on the experience of GenAI-mediated practice.

### 3.1 Participants

The participants were 70 undergraduate students enrolled in a Translation and Interpreting (T&I) program at a comprehensive university in China. All participants shared the same linguistic background, with Mandarin Chinese as their first language (L1) and English as their second language (L2).

Before the intervention, all students had completed foundational coursework in consecutive interpreting, but none had received systematic instruction in liaison interpreting or in the use of Generative AI for interpreting practice. Following a quasi-experimental design based on two intact classes, one class was designated as the Experimental Group (EG, *n* = 35) and the other as the Control Group (CG, *n* = 35). The EG included 22 female students and 13 male students, with a mean age of 20.5 years. The CG included 24 female students and 11 male students, with a mean age of 20.3 years. Independent samples t tests based on the previous semester’s interpreting grades showed no significant difference between the two groups (*p* > 0.05), indicating comparable baseline proficiency.

Because the two classes were pre-assigned by the university’s academic office for administrative purposes, random assignment of individual students was neither practically feasible nor ethically permissible, as it would have required disrupting existing class structures mid-semester. This intact-group design introduces the possibility of selection bias: unmeasured variables such as prior technology experience, motivation, or learning style preferences may have differed between the two classes. Moreover, because only one intact class was assigned to each condition, the design confounds treatment effects with any pre-existing class-level characteristics (e.g., group dynamics, peer composition, or classroom climate), making it impossible to isolate the effect of the instructional condition from that of the class itself. In addition, individual-level statistical tests treat the 70 students as independent observations, yet students within the same class share instructional context and social environment, potentially violating the independence assumption and underestimating standard errors. The between-group comparisons should therefore be interpreted as exploratory rather than confirmatory.

Several measures were taken to mitigate threats to internal validity beyond establishing baseline equivalence. First, the two groups followed an identical syllabus and were taught by the same instructor throughout the intervention. Second, the first 45 min of each weekly session were identical in content and delivery for both groups, ensuring equivalent instructional input. Third, the assignment of experimental and control conditions to the two classes was determined prior to any data collection. Fourth, interpreting performance was assessed using a double-blind rating procedure with anonymized and randomized recordings, preventing rater knowledge of group membership from influencing scores. Fifth, ANCOVAs with pretest scores as covariates were used to statistically adjust for any remaining pre-existing differences. Despite these precautions, the quasi-experimental design, particularly the assignment of only one class per condition, constitutes a fundamental constraint on causal inference, and all between-group comparisons should be regarded as exploratory, as further acknowledged in the Limitations section. Participation was entirely voluntary, and informed consent was obtained from all students before data collection in accordance with the university’s ethical guidelines.

To address the power asymmetry inherent in the instructor–researcher dual role, consent forms emphasized that participation was voluntary and that declining would carry no academic penalty; consent was collected by a research assistant rather than the instructor. Students were informed that their performance data would be anonymized before analysis and that no individually identifiable information would appear in any publication. Regarding the processing of voice data, students used the Doubao Voice Mode application on their personal devices; voice recordings and interaction transcripts generated during practice sessions were stored locally on students’ devices and were exported to the research team only with participants’ explicit consent. After transcription and coding were completed, all audio files were deleted from the research team’s storage. The research team did not have access to students’ personal Doubao accounts or usage data beyond the exported transcripts. Students were also informed that the commercial platform’s own data processing was governed by ByteDance’s privacy policy, which they could review independently.

### 3.2 Experiment process

The intervention was implemented over 8 weeks as part of a regular liaison interpreting course. As shown in Figure 1, the procedure consisted of three phases: preparation, thematic intervention, and evaluation.

**Figure 1:**
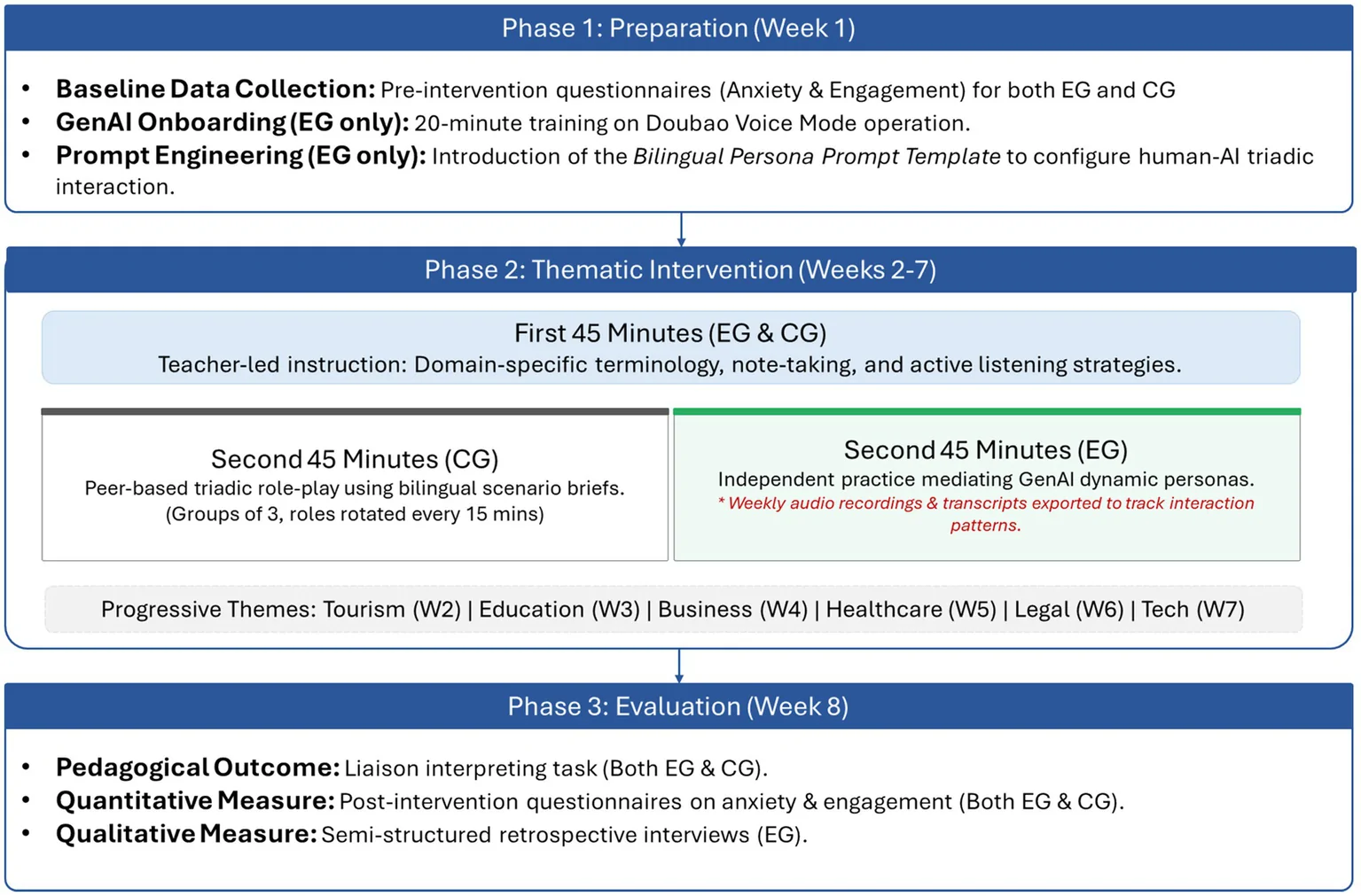
The experiment process.

*Phase 1: Preparation (Week 1)*. In the first week, baseline data were collected. All participants completed a liaison interpreting pretest and preintervention questionnaires measuring interpreting anxiety and learning engagement. Afterward, students in the EG attended a 20 min orientation on how to use the GenAI tool, Doubao Voice Mode, on their mobile devices. Doubao Voice Mode is a voice-enabled conversational application developed by ByteDance, powered by its Doubao large language model (version 1.8). The application was accessed on students’ personal mobile devices. System parameters were set to platform defaults and remained constant throughout the eight-week intervention. During this session, the instructor introduced the bilingual persona prompt template (see Appendix A) and demonstrated how the AI could be prompted to simulate two distinct interlocutors, such as an English speaking client and a Chinese speaking representative, thereby creating a human AI communicative triad.

*Phase 2: Thematic intervention (Weeks 2 to 7)*. During the following 6 weeks, both groups attended one 90 min session each week. The sessions focused on progressively more complex themes: Tourism and Hospitality (Week 2), Education and Community (Week 3), Business Negotiation (Week 4), Healthcare Consultation (Week 5), Legal Assistance (Week 6), and Technology and Environment (Week 7).

The first 45 min of each session were identical for both groups and consisted of instructor-led teaching on domain specific terminology, note taking, and active listening strategies. The remaining 45 min formed the active practice stage. In the CG, students worked in groups of three. Based on bilingual scenario briefs provided by the instructor, two students performed the roles of the primary interlocutors and the third served as the interpreter. Roles were rotated every 15 min to ensure equal practice time. In the EG, students practiced individually with the GenAI tool. By using prompt templates designed for each weekly theme, students configured the AI to alternate dynamically between the two interlocutor roles. Each student acted as the sole human interpreter and was responsible for mediating the interaction, managing turn taking, and resolving communication breakdowns in real time. It should be noted that this design resulted in an asymmetry in direct interpreting time: because CG students rotated among three roles every 15 min, each student spent approximately 15 min per session in the interpreter role, whereas EG students interpreted continuously, for the full 45-min practice period—approximately three times as much direct interpreting practice per session (roughly 270 versus 90 min cumulatively across the six intervention weeks). In addition, the two conditions differed in practice format: EG students practiced individually, while CG students practiced in triadic groups. These asymmetries were not controlled experimentally; rather, they reflect the inherent structural differences between GenAI-mediated individual practice and conventional peer role-play as each would be implemented in authentic pedagogical settings. Equalizing direct interpreting time would have required either artificially constraining GenAI use or restructuring peer role-play in ways that compromise the ecological validity of both conditions. Nevertheless, because practice dosage and practice format co-vary with the instructional condition, they constitute potential confounds that limit the attributability of any observed differences to the GenAI medium alone (see the Limitations section, where this practice-time asymmetry is identified as the central limitation of the study). To trace the development of human AI interaction over time, students in the EG were required to audio record their weekly practice sessions and export the transcripts for analysis.

To document intervention fidelity, the following information is reported. All 35 EG students and all 35 CG students completed all six intervention sessions (Weeks 2–7); no sessions were missed. Each 45-min practice period was timed by the instructor to ensure comparable duration across groups. For the EG, the mean number of interpreted turns per student per session was approximately 20.73 (SD = 1.79), derived from the coded transcripts. The GenAI tool (Doubao Voice Mode, version 1.8) was accessed on students’ personal mobile devices throughout the intervention period; system parameters were set to platform defaults and remained unchanged. Weekly persona prompt templates and CG scenario briefs were prepared by the instructor and are available in Appendix A; the same instructor delivered all sessions for both groups. Observed AI malfunctions included occasional language switching, persona inconsistency, and factually inconsistent output (see Section 4.4.2, Theme 2); these were managed by students in real time and were not systematically logged. As reported in Section 4.4.1, some EG students engaged in self-initiated practice outside class, but no exposure logs were collected (see Limitations).

*Phase 3: Evaluation (Week 8)*. In the final week, all participants completed a posttest liaison interpreting task and postintervention questionnaires on anxiety and learning engagement. To complement the quantitative data, semi structured retrospective interviews were conducted with eight EG students selected purposively to represent different levels of interpreting proficiency. The interviews explored students’ perceptions of the affordances of GenAI, the challenges they encountered, and the ways in which they exercised learner agency during immersive dialogic practice.

### 3.3 Instruments

Students’ interpreting anxiety was measured with an adapted version of the Foreign Language Classroom Anxiety Scale (FLCAS), originally developed by Horwitz et al. (1986) and later tailored to interpreting contexts by Chiang (2010). The original English items were adapted to reflect the triadic, dialogue-based nature of liaison interpreting (e.g., replacing references to “foreign language class” with “interpreting role-play”) and then translated into Chinese using a back-translation procedure: two bilingual researchers independently translated the adapted items into Chinese, a third researcher back-translated the Chinese version into English, and discrepancies between the back-translation and the original were resolved through discussion. The translated instrument was pilot-tested with 15 interpreting students from a separate cohort to check item clarity; minor wording adjustments were made based on their feedback. The adapted scale contains 12 items designed to assess anxiety in dialogic interpreting tasks. It covers three dimensions: communication apprehension (e.g., “I start to panic when I have to interpret a complex sentence without preparation”), fear of negative evaluation (e.g., “I am afraid the other interlocutors will notice my interpreting mistakes”), and general performance anxiety (e.g., “Even if I am well prepared, I feel anxious about role playing as an interpreter”). All items were rated on a five-point Likert scale. In the present study, the scale demonstrated high internal consistency (Cronbach’s alpha = 0.91).

Learning engagement was measured using the Utrecht Work Engagement Scale for Students, developed by Schaufeli et al. (2002). Items were translated into Chinese following the same back-translation procedure described above and were included in the same pilot test, which confirmed item clarity. This 14 item instrument conceptualizes engagement as a positive, fulfilling, study related state of mind comprising three dimensions: vigor (e.g., “When practicing interpreting, I feel mentally resilient”), dedication (e.g., “I find the interpreting role play sessions full of meaning and purpose”), and absorption (e.g., “Time flies when I am engaged in the interpreting practice”). The absorption dimension was especially relevant to the present study because it reflects the degree to which learners become immersed in the GenAI-mediated dialogic environment. All items were rated on a five-point Likert scale. In the present study, the instrument showed strong reliability (Cronbach’s alpha = 0.89).

### 3.4 Data collection and analysis

#### 3.4.1 Liaison interpreting performance

To answer RQ1, students’ pretest and posttest liaison interpreting performances were analyzed based on audio recordings. Performance was assessed with a multidimensional analytic rubric adapted from established interpreting assessment frameworks (Hale, 2007; Pöchhacker, 2022) to reflect the interactional demands of liaison interpreting. The rubric included three weighted dimensions: (1) Informational Accuracy and Completeness (50%), which assessed the faithful transfer of source messages without unjustified omissions or distortions; (2) Target Language Quality and Delivery (20%), which evaluated linguistic accuracy, fluency, and pacing; and (3) Interactional Management (30%), which examined the interpreter’s ability to coordinate the communicative triad, manage turn taking, use clarification strategies, and respond appropriately to pragmatic shifts (Wadensjö, 2014). The complete rubric, including five-level performance descriptors and scoring anchors for each dimension, is provided in Appendix B.

The pretest and posttest tasks were designed as parallel forms of a triadic liaison interpreting scenario, matched on task format, number of turns, source-speech length, estimated completion time, distribution of communicative functions, and domain complexity. Neither scenario overlapped with the six thematic modules covered during the intervention. Both tasks were piloted with six interpreting students from a separate cohort in counterbalanced order, and no significant difficulty difference was found (paired-samples *t* test, *p* > 0.05). To reduce subjective bias, a double blind rating procedure was adopted. All audio recordings were anonymized and randomized, and any information revealing group membership or testing occasion was removed. Two experienced interpreting instructors, each with more than 5 years of teaching experience and no knowledge of the study’s specific hypotheses, served as independent raters. Before formal scoring, the raters completed a calibration session in which they jointly evaluated four sample recordings not included in the main dataset to align their interpretation of the rubric.

The two raters then scored all 140 recordings independently. Inter-rater reliability was assessed using intraclass correlation coefficients based on a two-way mixed model with consistency (single measures, ICC(3,1); Shrout and Fleiss, 1979). ICCs ranged from 0.884 to 0.919 across all dimensions and testing occasions, indicating excellent agreement (Cicchetti, 1994). All *F* tests were significant at *p* < 0.001. When their total scores differed by more than 10 points out of 100 (i.e., three cases), they discussed the case until a consensus score was reached. For all cases, the mean of the two raters’ scores was used as the final score. After scoring was completed, independent samples *t*-tests were first conducted on the pretest scores to verify baseline equivalence. One-way analyses of covariance (ANCOVAs) were then performed on the posttest scores, with the corresponding pretest score entered as a covariate to control for pre-existing individual differences. Assumptions of homogeneity of regression slopes, homogeneity of variance, and normality of residuals were evaluated prior to interpretation. No data were missing for any participant.

#### 3.4.2 Interpreting anxiety and learning engagement

To answer RQ2, independent samples t tests were first conducted on the pretest scores for anxiety and engagement to confirm baseline equivalence between the EG and the CG. ANCOVAs were then performed to examine the effects of the GenAI intervention. Two separate ANCOVA models were estimated, one for learning engagement and one for interpreting anxiety. In each model, the posttest score served as the dependent variable, instructional condition (EG vs. CG) served as the between subjects factor, and the corresponding pretest score was entered as the covariate.

#### 3.4.3 Interaction patterns

To answer RQ3, the transcripts of the human AI interactions were analyzed through a combined deductive and inductive approach. For this purpose, a specialized analytical framework, the Three Dimensional Dynamic Interpreting Interaction Coding Scheme (see Table 1), was developed for the study. The scheme draws on Wadensjö’s (2014) dialogic interpreting theory, Long’s (1996) framework of negotiation of meaning, and recent scholarship on learner agency in human AI collaboration (Tai and Chen, 2025).

**Table 1:** The coding scheme.

Dimension	Code	Definition	Example from GenAI-mediated practice
Meaning transfer and pragmatic adaptation	Close Rendition (CR)	Faithful and accurate transfer of the AI’s literal meaning without significant pragmatic or structural shifts.	AI (Doctor): “You need to take this pill twice a day.” Student: “你需要每天吃两次这个药。”
	Strategic Expansion/Reduction (SER)	Deliberate addition of explanatory background information or omission of redundant elements to facilitate target listener comprehension.	AI (Patient): “I feel a sharp pain here, like needles poking my stomach.” Student: “他这里有严重的刺痛感。” *(Omission of the metaphor for conciseness)*
	Pragmatic / Cultural Adaptation (PCA)	Adjusting the register, tone, or cultural references to mitigate pragmatic dissonance or cultural conflicts.	AI (Buyer): “Your price is absolutely ridiculous!” Student: “客户认为目前的价格可能超出了他们的预算范围.” *(Softening the aggressive tone)*
Interaction management and meaning negotiation	Clarification/Confirmation Request (CCR)	Interrupting the AI to ask for the repetition, explanation, or confirmation of ambiguous source utterances or specialized terminology.	Student: “Sorry doctor, could you please explain what ‘endoscopy’ means in simple words before I translate?”
	Turn-taking and Pacing Management (TPM)	Controlling the flow of the conversation, such as pausing the AI’s long speech or prompting the next interlocutor to speak.	Student: “Excuse me, please pause for a moment so I can translate this part for the patient first.”
	Breakdown Repair (BR)	Intervening to correct the interpreter’s own previous translation errors or resolving logical misunderstandings between the two AI personas.	Student: “抱歉, 我刚才翻译有误, 医生说的不是胃溃疡, 而是.” *(Correcting a previous interpreting mistake)*
Human-AI collaboration and learner agency	Persona/Prompt Adjustment (PPA)	Stepping out of the interpreter role to issue natural language meta-prompts that fine-tune the AI’s behavior, tone, or persona constraints.	Student: “你刚才的语气太正式了, 请你现在表现得更像一个焦急、说话口语化的家属。” *(Prompting the AI to adjust its persona)*
	Hallucination and Glitch Handling (HGH)	Strategies deployed to cope with AI technical failures, such as language confusion, memory lapses, or role inconsistency.	AI: *(Suddenly speaks English while playing the Chinese doctor)* Student: “Please switch back to your role as the Chinese doctor and speak Mandarin.”
	Socio-affective Alignment (SAA)	Demonstrating genuine emotional resonance, empathy, or tension in response to the AI’s simulated affective states, indicating a high level of immersion.	Student: *(Using a soothing tone and sighing)* “Please do not worry too much, the doctor said it is curable.”

The framework contains three main dimensions. Meaning Transfer and Pragmatic Adaptation captures how students processed and adapted the bilingual information generated by the AI and corresponds to Wadensjö’s notion of renditions. Interaction Management and Meaning Negotiation focuses on non renditions and examines how learners managed the triadic exchange, requested clarification, and repaired communication breakdowns. Human AI Collaboration and Learner Agency was developed specifically for GenAI-mediated settings. It captures how students exercised agency by refining AI personas, handling technical problems or hallucinations, and responding to the AI’s simulated affective cues.

Using this coding scheme, two researchers independently coded a randomly selected 20% subset of the transcripts. The initial interrater reliability analysis yielded a Cohen’s kappa of 0.85, indicating strong agreement. The researchers then discussed all discrepancies until full consensus was reached. After this calibration stage, the remaining transcripts were coded independently. A final interrater reliability check was conducted after coding, and any remaining disagreements were resolved through discussion to produce the final dataset for network analysis.

To characterize the structural connections among students’ interactional strategies, the finalized coded dataset was analyzed through Epistemic Network Analysis (ENA), using the ENA Web Tool (version 2.0.7, epistemicnetwork.org). Situated within the tradition of quantitative ethnography, ENA is designed to quantify and visualize the co-occurrence of coded behaviors (Shaffer et al., 2016; Wang et al., 2025). Unlike conventional frequency based approaches that treat codes as isolated events, ENA models the relational strength and structural connections among discourse elements within an interaction (Tan et al., 2024).

The ENA model was specified using four parameters. Codes referred to the annotations assigned to students’ utterances according to the coding scheme. Conversation referred to the sequence of continuous exchanges between the student interpreter and the GenAI within a single thematic practice session. The unit of analysis was the individual student in each week. The stanza was defined as a moving window of eight conversational lines. A sensitivity analysis was conducted comparing window sizes of 4, 5, 6, 7, and 8 lines; a window of 8 was selected because it yielded the most interpretable and stable network structure while remaining consistent with the mean turn-exchange length observed in the data. Networks were normalized using sphere normalization, and the dimensional reduction was performed using singular value decomposition (SVD), both of which are the platform defaults. All 35 EG students contributed data from all six intervention sessions (Weeks 2–7); no sessions were missing. The resulting model is an aggregate, descriptive representation of co-occurrence patterns across the full intervention period; no week-by-week or longitudinal comparison was conducted.

#### 3.4.4 Interview

To answer RQ4, semi-structured interviews were conducted with eight EG students by the first author, who had served as the course instructor for both groups throughout the intervention. Although this dual role may have introduced social-desirability bias, it also enabled rapport and contextual understanding that facilitated in-depth discussion. To mitigate power asymmetry, interviews were conducted after all course grades had been finalized, and students were assured that their responses would not affect their academic standing. Participants were selected purposively to represent a range of posttest interpreting performance levels (two high, four mid, two low) in order to capture diverse experiences. Each interview was conducted in Mandarin Chinese, lasted approximately 20–30 min, and was audio-recorded with the participant’s consent. Recordings were transcribed verbatim in Chinese by a research assistant and checked for accuracy by the first author. Illustrative quotations presented in the manuscript were translated into English by the first author and verified by a bilingual colleague. Eight participants were deemed sufficient to achieve data adequacy for this focused research question, as thematic saturation—the point at which no new themes or subthemes emerged—was reached by the seventh interview.

The transcripts were analyzed using reflexive thematic analysis (Braun and Clarke, 2006). Coding followed an iterative approach that combined deductive and inductive procedures. The deductive phase was guided by existing theoretical constructs related to technological affordances and sociocultural agency, whereas the inductive phase allowed new participant driven insights to emerge concerning the constraints of GenAI in interpreting practice. Initial codes were systematically compared, refined, and grouped into broader themes, such as perceived authenticity of simulation, frustration with technological hallucinations, and proactive prompt negotiation. Peer debriefing within the research team was used to strengthen the trustworthiness, reflexivity, and interpretive rigor of the final thematic structure. The first author acknowledges a potential confirmatory bias arising from the dual instructor–researcher role; to address this, coding was reviewed independently by the second author, who had no involvement in the teaching, and discrepancies were resolved through discussion.

## 4 Results

### 4.1 Liaison interpreting performance

To explore the association between GenAI-supported role play and students’ liaison interpreting performance, pretest and posttest scores were compared between the experimental and control groups. Given that class-level assignment precludes definitive causal attribution (see Section 3.1), these comparisons are exploratory.

Independent samples *t*-tests were first conducted on pretest scores to examine baseline equivalence. As shown in Table 2, no significant differences were found between the two groups in informational accuracy, *t*(68) = 0.02, *p* = 0.985; target language quality, *t*(68) = −1.33, *p* = 0.188; interactional management, *t*(68) = −1.43, *p* = 0.157; or total score, *t*(68) = −1.28, *p* = 0.205. These results confirm that the two groups exhibited comparable interpreting performance prior to the intervention.

**Table 2:** The results of independent samples t-tests comparing pretest interpreting performance between the EG and CG.

Dimension	EG (*n* = 35)	CG (*n* = 35)	*t(68)*	*p*	95% CI
Informational accuracy	34.06 (4.61)	34.04 (5.28)	0.02	0.985	[−2.34, 2.39]
Target Language quality	12.42 (2.36)	13.22 (2.65)	−1.33	0.188	[−1.99, 0.40]
Interactional management	18.76 (3.42)	19.96 (3.60)	−1.43	0.157	[−2.87, 0.47]
Total score	65.25 (6.11)	67.22 (6.77)	−1.28	0.205	[−5.05, 1.10]

CI = 95% confidence interval of the mean difference (Experimental − Control). None of the pre-test comparisons reached statistical significance (all ps > 0.05).

To further control for any pre-existing individual differences, a one-way analysis of covariance (ANCOVA) was conducted for each dimension and the total score, with the corresponding pretest score entered as a covariate. As shown in Table 3, after adjusting for pretest performance, the experimental group scored significantly higher than the control group in overall interpreting performance (adjusted *M* = 79.05 vs. 73.54), *F*(1, 67) = 8.50, *p* = 0.005, partial *η*^2^ = 0.113, indicating a medium-to-large effect.

**Table 3:** The ANCOVA results comparing posttest interpreting performance between the EG and CG with pre-test as covariate.

Dimension	Adjusted mean		*F(1, 67)*	*p*	Partial *η*^2^	95% CI
	EG	CG				
Informational accuracy	39.39	36.74	4.15	0.046*	0.058	[0.05, 5.26]
Target language quality	15.65	15.18	0.73	0.395	0.011	[−0.62, 1.56]
Interactional management	24.02	21.62	6.79	0.011*	0.092	[0.56, 4.24]
Total score	79.05	73.54	8.50	0.005**	0.113	[1.74, 9.29]

Adjusted means are evaluated at the grand mean of the pre-test covariate. CI = 95% confidence interval of the adjusted mean difference (Experimental − Control). *p < 0.05. **p < 0.01. ***p < 0.001.

At the level of individual dimensions, significant between-group differences were found for informational accuracy, (adjusted *M* = 39.39 vs. 36.74), *F*(1, 67) = 4.15, *p* = 0.046, partial *η*^2^ = 0.058, and interactional management, (adjusted *M* = 24.02 vs. 21.62), *F*(1, 67) = 6.79, *p* = 0.011, partial *η*^2^ = 0.092, with the experimental group scoring higher than the control group on both dimensions. The effect size for interactional management was medium, whereas that for informational accuracy was small to medium. By contrast, no significant between-group difference was observed for target language quality, (adjusted *M* = 15.65 vs. 15.18), *F*(1, 67) = 0.73, *p* = 0.395, partial *η*^2^ = 0.011, suggesting that the two instructional conditions produced comparable outcomes in linguistic output.

Taken together, these findings suggest that, even after controlling for pretest performance, GenAI-supported role play was associated with higher posttest performance than traditional peer role-play, particularly with respect to information transfer and interaction coordination. However, because only one class was assigned to each condition and EG students accumulated approximately three times as much direct interpreting practice as CG students, this pattern cannot be attributed solely to the GenAI medium: the advantage may reflect the greater volume of direct interpreting practice rather than the practice modality itself.

### 4.2 Interpreting anxiety and learning engagement

To assess the effects of GenAI-supported role play on interpreting related anxiety and learning engagement, analyses of covariance were conducted with posttest scores as the dependent variables and pretest scores as the covariates. Before the intervention, the two groups did not differ significantly in either interpreting anxiety, t(68) = −0.228, *p* = 0.821, or learning engagement, t(68) = −0.587, *p* = 0.559, thus confirming baseline equivalence on both measures.

For interpreting related anxiety, the ANCOVA revealed a significant between group difference after controlling for pretest scores, *F*(1, 67) = 21.185, *p* < 0.001, partial η^2^ = 0.240. The experimental group reported substantially lower posttest anxiety (adjusted M = 2.51) than the control group (adjusted M = 2.96). The effect size was large, indicating that GenAI-supported role play was associated with considerably lower interpreting anxiety than traditional peer role-play.

For learning engagement, however, the ANCOVA showed no significant between group difference, *F*(1, 67) = 2.368, *p* = 0.129, partial η^2^ = 0.034. Although the experimental group obtained a slightly higher adjusted mean (3.65) than the control group (3.48), the difference did not reach statistical significance. This result suggests that the two instructional approaches generated comparable levels of learning engagement over the intervention period.

Overall, these exploratory findings indicate that GenAI-supported role play was associated with significantly lower interpreting related anxiety relative to traditional peer role-play, whereas the difference in learning engagement was not statistically significant (Tables 4–7).

**Table 4:** The independent samples *t*-test comparing interpreting anxiety in the pretest.

Group	*n*	Mean	SD	*t*	*p*
EG	35	3.08	0.47	−0.228	0.821
CG	35	3.11	0.46		

**Table 5:** The ANCOVA results comparing interpreting anxiety between groups with pretest as covariate.

Group	*n*	Pretest mean (SD)	Posttest mean (SD)	Adjusted mean	F	p	Partial η^2^	95% CI
EG	35	3.08 (0.47)	2.50 (0.48)	2.51	21.185	<0.001	0.240	[−0.65, −0.26]
CG	35	3.11 (0.46)	2.97 (0.53)	2.96				

**Table 6:** The independent samples *t*-test comparing learning engagement in the pretest.

Group	*n*	Mean	SD	*t*	*p*
EG	35	3.35	0.49	−0.587	0.559
CG	35	3.42	0.54		

**Table 7:** The ANCOVA results comparing learning engagement between groups with pretest as covariate.

Group	*n*	Pretest mean (SD)	Posttest mean (SD)	Adjusted mean	*F*	*p*	Partial η^2^	95% CI
Experimental	35	3.35 (0.49)	3.63 (0.53)	3.65	2.368	0.129	0.034	[−0.05, 0.38]
Control	35	3.42 (0.54)	3.51 (0.54)	3.48				

### 4.3 Interaction patterns

To characterize students’ interaction patterns and meaning negotiation strategies during GenAI-supported role play, the coded transcripts were first examined descriptively and then modeled using ENA. The results presented below reflect the aggregate structural profile of interaction across the six-week intervention period and do not represent a developmental trajectory.

#### 4.3.1 Frequency distribution of interaction strategies

As shown in Figure 2, Close Rendition (CR) was the most frequently occurring code across all sessions, indicating that students’ primary task was to transfer meaning faithfully between the two AI interlocutors. To make the distribution of less routine strategies more visible, Figure 2 presents the frequencies after CR was excluded. Among the remaining codes, Clarification or Confirmation Request (CCR) was the most prevalent strategy (*n* = 300, 49.9%), followed by Strategic Expansion or Reduction (SER; n = 123, 20.5%) and Breakdown Repair (BR; n = 112, 18.6%). Pragmatic or Cultural Adaptation (PCA) accounted for 9.5% (*n* = 57). In contrast, codes related to human and AI collaboration and learner agency occurred only rarely. Specifically, Hallucination & Glitch Handling (HGH; n = 4, 0.7%), Socioaffective Alignment (SAA; *n* = 2, 0.3%), Turn Taking & Pacing Management (TPM; n = 2, 0.3%), and Persona or Prompt Adjustment (PPA; *n* = 1, 0.2%) together represented less than 2% of all non CR codes.

**Figure 2:**
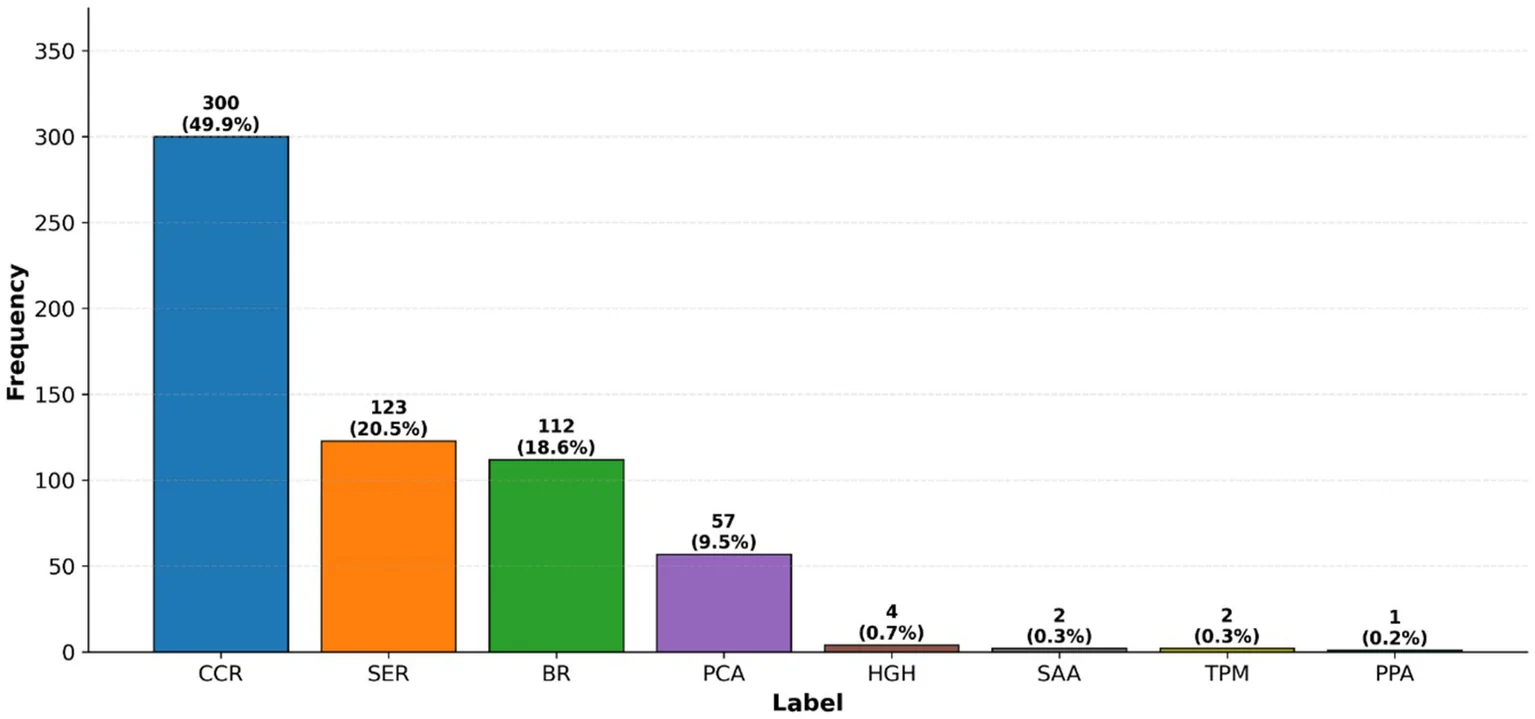
Frequency distribution of students’ interaction strategies in the EG.

These frequency patterns suggest that students’ GenAI-mediated interpreting practice was primarily characterized by meaning transfer, supplemented by active clarification seeking and self repair. By comparison, explicit metacognitive regulation of the AI system appeared to be relatively uncommon.

#### 4.3.2 Epistemic network analysis of co-occurrence patterns

The ENA model shown in Figure 3 illustrates the aggregate structural connections among coded behaviors across the intervention period, with individual students treated as the units of analysis nested within weekly sessions. The first two dimensions of the SVD accounted for 45.21 and 30.25% of the variance. Node size represents the relative frequency of each code, whereas edge thickness indicates the strength of co-occurrence between code pairs within the defined stanza window.

**Figure 3:**
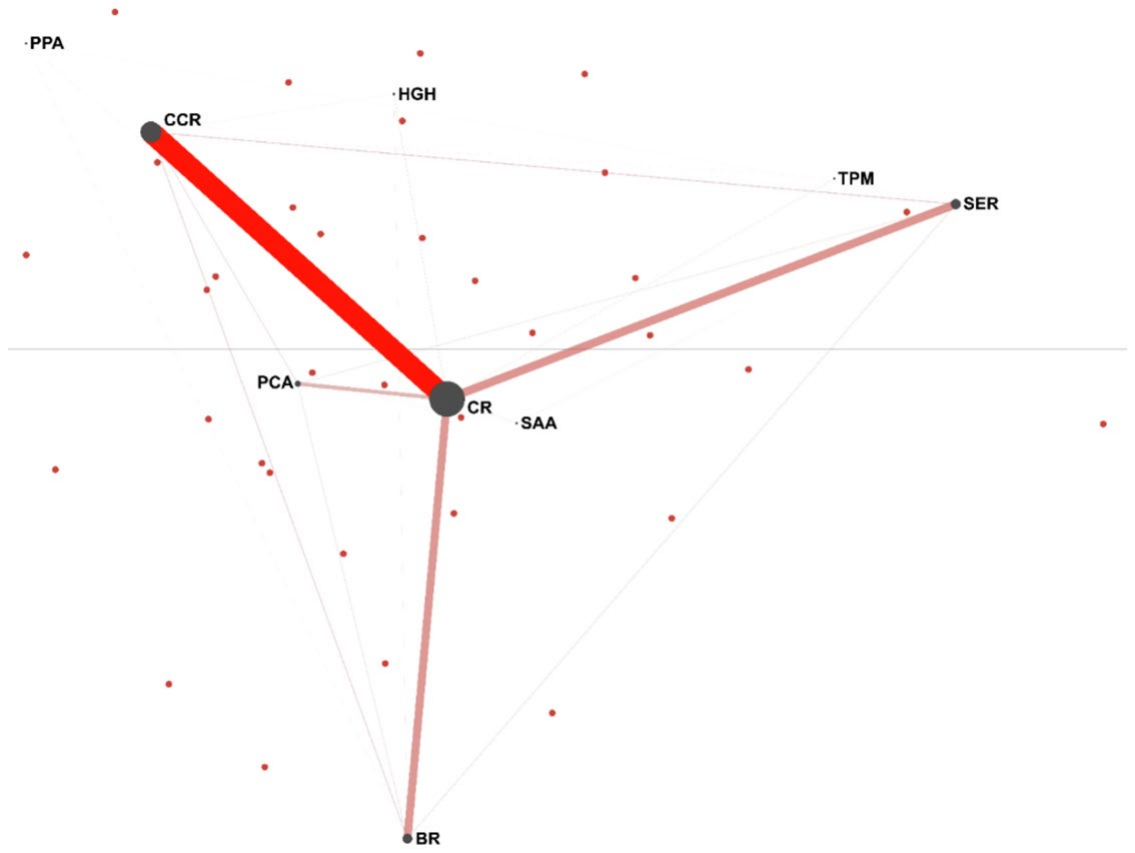
Aggregate mean network of co-occurrence patterns for students in the EG. Nodes represent coded interaction strategies; edge thickness reflects the relative co-occurrence weight between code pairs. Individual points represent student-level network centroids.

CR occupied the central position in the network, with the strongest connections extending outward from this node. The most prominent edge linked CR and CCR (edge weight = 0.83), indicating that faithful renditions and clarification requests most frequently cooccurred within the same stanza window. The second strongest connection was observed between CR and BR (edge weight = 0.31), suggesting that breakdown repair regularly accompanied meaning transfer activities. A third notable connection linked CR and SER (edge weight = 0.32), reflecting the frequent co-occurrence of close renditions with strategic expansion or reduction of information.

A moderately weighted connection was also observed between CR and PCA (edge weight = 0.15). Weaker but still visible connections linked CR with TPM and SAA. The codes associated with human and AI collaboration, namely PPA, HGH, and SAA, were positioned at the periphery of the network and exhibited minimal edge weights, suggesting weak connections both with the central meaning transfer node and with one another.

Overall, the ENA network displayed a hub and spoke structure centered on CR. Within this structure, CCR, BR, and SER formed the most strongly connected nodes, whereas interaction management and human and AI collaboration codes remained structurally peripheral.

### 4.4 Students’ perception

Reflexive thematic analysis of the semi-structured interview transcripts identified three overarching themes comprising twelve subthemes. These themes were: (1) perceived affordances of GenAI as an interactive partner, (2) perceived limitations and constraints of GenAI, and (3) learner agency in the GenAI-mediated environment.

#### 4.4.1 Theme 1: perceived affordances of GenAI as an interactive partner

The first theme captured students’ positive perceptions of what the GenAI tool enabled them to accomplish in their interpreting practice. Four subthemes were identified.

The first subtheme, a low anxiety and psychologically safe practice space, referred to students’ perception of AI as a nonjudgmental interlocutor that reduced the performance anxiety typically associated with practicing in front of peers or instructors. As S3 explained, “When I make mistakes in front of classmates, I feel embarrassed. But with the AI, I can mess up ten times and nobody cares. It just keeps going.”

The second subtheme, unlimited repetition and patience, captured students’ appreciation of the AI’s capacity to repeat utterances, adjust speech rate, or restart scenarios without displaying impatience or frustration. S6 described this affordance as follows: “I asked it to repeat the same sentence maybe five or six times because I could not catch the medical term. A real person would get annoyed, but the AI just repeated it every time.”

The third subtheme, on demand availability and practice autonomy, referred to the flexibility students experienced in arranging practice at self selected times and for self determined durations beyond the formal classroom setting. Because no systematic exposure logs were collected, the frequency and duration of such extracurricular practice could not be quantified, and this unmonitored additional exposure constitutes a further confound when interpreting the between-group comparisons (see Limitations). As S1 noted, “I sometimes practiced at midnight before sleeping. I could do an extra 20 min session whenever I wanted without coordinating schedules with partners.”

The fourth subtheme, perceived realism and immersiveness of simulation, described students’ view that the AI’s ability to sustain two distinct interlocutor personas created a sense of communicative authenticity that facilitated deep task immersion. S5 reported, “After a few minutes I almost forgot it was AI. The doctor character sounded professional and the patient sounded confused and worried, it felt like a real consultation.”

#### 4.4.2 Theme 2: perceived limitations and constraints of GenAI

The second theme captured the challenges and frustrations students encountered when using GenAI as a practice partner. Four subthemes emerged.

The first subtheme, role confusion and hallucinations, referred to instances in which the AI unexpectedly switched languages, departed from its assigned character, or generated factually inconsistent information, thereby disrupting the flow of practice. S2 explained, “Sometimes the ‘Chinese doctor’ suddenly spoke English for no reason, or the patient mentioned symptoms that contradicted what was said 2 min earlier.”

The second subtheme, absence of authentic nonverbal and paralinguistic cues, reflected students’ observation that the AI lacked facial expressions, gestures, and embodied communication, which constrained their ability to make pragmatic judgments about interlocutors’ comprehension. S7 reflected, “In real interpreting you can read body language to know if someone is confused. With the AI, I only had the voice, so I sometimes could not tell if my translation was understood.”

The third subtheme, unnatural turn taking and pacing, described students’ perception that the conversational rhythm of the AI occasionally felt mechanical, either because it responded too quickly and left insufficient processing time or because it produced excessively long monologues without natural pauses. As S4 noted, “It sometimes gave me a 3 min speech without pausing. In real life, people would naturally stop and wait for the interpreter.”

The fourth subtheme, limited evaluative feedback on interpreting quality, referred to students’ frustration that the AI could not assess the accuracy or appropriateness of their renditions or provide corrective feedback on errors. S8 stated, “It never told me if my translation was right or wrong. I could say something completely inaccurate and it would just continue the conversation as if nothing happened.”

#### 4.4.3 Theme 3: learner agency in the GenAI-mediated environment

The third theme captured the agentic strategies students employed to optimize their learning within the GenAI-mediated environment. Four subthemes were identified.

The first subtheme, proactive prompt negotiation and persona refinement, described students’ practice of actively modifying prompts during a session in order to adjust the AI’s speech rate, register, or persona characteristics to better match their desired practice conditions. S5 provided the following example: “I told it: ‘Speak faster and use more colloquial expressions because the patient should sound like an elderly rural person.’ It changed immediately and the practice felt more realistic.”

The second subtheme, self directed difficulty scaffolding, referred to students’ deliberate adjustment of task complexity through changes in scenario parameters such as topic technicality, speech speed, or interlocutor emotionality. S1 described this progression: “In the first few weeks I kept scenarios simple. By Week 5, I started telling the AI to use more jargon and interrupt me, because I wanted more pressure.”

The third subtheme, strategic coping with AI breakdowns, captured the range of corrective strategies students developed to manage hallucinations or role inconsistencies rather than abandoning the task. As S3 explained, “When it broke character, I learned to say something like ‘Please remember you are the patient and continue in Chinese.’ After a while I got good at pulling it back on track quickly.”

The fourth subtheme, reflective self monitoring and post session review, described students’ practice of reviewing their own recordings or transcripts after practice sessions in order to identify recurring errors and set improvement goals for subsequent sessions. S6 reported, “Every weekend I re read the transcripts and highlighted where I hesitated or mistranslated. Then next week I focused specifically on those weak points.”

In summary, the thematic analysis showed that students perceived GenAI as a psychologically safe, patient, and flexibly available practice partner capable of generating immersive simulations. At the same time, they identified role instability, the absence of nonverbal cues, unnatural pacing, and limited corrective feedback as important constraints. In response to both the affordances and the limitations of the system, students exercised agency through proactive prompt negotiation, self directed difficulty management, strategic repair of AI breakdowns, and reflective self monitoring.

## 5 Discussion

### 5.1 General discussion

The present study investigated the effectiveness of GenAI-mediated immersive dialogue practice in a university-level liaison interpreting course. Before discussing the theoretical implications of the findings, an important caveat is warranted. The present study measured interpreting performance, interpreting anxiety, learning engagement, and interaction patterns; it did not directly assess the cognitive and motivational constructs—cognitive load, working-memory allocation, schema development, and self-efficacy—that are invoked below to interpret the results. All mechanistic explanations offered in this section are therefore *post hoc* theoretical accounts that are consistent with the observed data but remain to be tested through dedicated measures (e.g., dual-task paradigms for cognitive load, self-efficacy scales, think-aloud protocols for schema use). Overall, the findings indicate that GenAI can function as a psychologically facilitative interactive partner. More specifically, it appears to be associated with higher scores on selected dimensions of interpreting competence and lower levels of affective barriers to practice, while also introducing constraints that call for careful design of the practice environment. However, as discussed below, several design confounds—including differences in practice dosage, practice format, and unmonitored extracurricular exposure—preclude definitive attribution of these patterns to the GenAI medium alone.

With regard to interpreting performance (RQ1), the experimental group scored significantly higher than the control group in overall scores after controlling for pretest performance, with particularly clear differences in informational accuracy and interactional management. Because this comparison involves only one class per condition, the pattern should be interpreted as exploratory and may partly reflect pre-existing class-level differences rather than the instructional treatment alone. This finding is consistent with the predictions of Cognitive Load Theory (Sweller et al., 2011), which holds that practice environments generating intrinsic cognitive load profiles that approximate authentic professional demands are more effective in promoting schema acquisition and automation. The GenAI tool’s ability to generate register-appropriate, terminologically dense, and contextually varied interlocutor discourse likely provided more cognitively authentic input than that typically available in peer role-play, where interlocutor performance is often simplified and register-inconsistent (Sandrelli and de Manuel Jerez, 2007; Qu and Chen, 2026). By exposing learners to sustained, complex input, such practice may have facilitated the development of more robust interpreting schemas—automated cognitive structures enabling rapid recognition and processing of recurrent communicative patterns (van Merriënboer and Sweller, 2005). However, schema development was not directly measured in this study, and this interpretation remains a theoretically motivated hypothesis rather than an empirical finding. The significant gain in interactional management is also consistent with Wadensjö's (2014) dialogic perspective: the unpredictability and dynamism of AI-generated turn exchange may have created conditions that demanded greater executive control**—**including inhibition, cognitive flexibility, and self-monitoring (Miyake et al., 2000; Diamond, 2013)—that are central to real-time coordination. Whether such executive processes were in fact strengthened cannot be determined from the present data. However, these performance differences must be interpreted with caution because the two conditions differed not only in the source of interlocutor input but also in practice dosage and practice format. EG students interpreted continuously for 45 min per session, whereas CG students interpreted for approximately 15 min due to role rotation—approximately three times as much direct interpreting practice—and the individual-versus-triadic practice format constitutes an additional confound. Furthermore, the interview data indicate that some EG students engaged in self-initiated practice outside class, but no exposure logs were collected to quantify this additional input. It is therefore not possible to determine whether the observed performance advantages reflect the GenAI medium specifically, the greater volume of direct interpreting practice, the individual practice format, extracurricular exposure, or some combination of these factors. By contrast, the absence of a significant between-group difference in target language quality suggests that output-related features such as fluency, lexical choice, and grammatical control may depend more heavily on learners’ broader second-language proficiency than on the specific practice modality. From an embodied cognition perspective (Danesi, 2017; Shapiro and Stolz, 2019), the voice-only modality of the AI tool may also have limited opportunities for the multimodal, gesture-embedded processing that contributes to conceptual fluency in face-to-face interpreting. This interpretation is consistent with prior research showing that productive linguistic competence tends to develop gradually and is less susceptible to short-term intervention (Lu, 2025).

The findings on anxiety (RQ2) further showed that GenAI-supported practice was associated with significantly lower interpreting-related anxiety relative to traditional peer role-play, and the effect size was large. This result can be interpreted through Eysenck and Calvo's (1992) processing efficiency theory: by minimizing the evaluative social presence associated with classmates and instructors, GenAI-mediated practice likely reduced the worry-related cognitive processing that competes with task-relevant processing for working memory resources (Horwitz et al., 1986). It should be noted, however, that working-memory allocation was not directly measured; this interpretation is a theoretically plausible account of the observed anxiety reduction, not a demonstrated mechanism. The interview data reinforce this interpretation, as students explicitly identified the AI’s non-judgmental stance and unlimited patience as key features that made practice less stressful. From the perspective of Self-Efficacy Theory (Bandura, 1997), a lower-threat practice environment may have enabled learners to engage with more challenging scenarios and experience more frequent enactive mastery—theoretically the most powerful source of self-efficacy development. Self-efficacy was not measured in this study, so this pathway remains hypothetical. In this sense, anxiety reduction may have served as a cognitive-affective catalyst: by hypothetically freeing working memory resources and fostering mastery experiences, it may have created conditions conducive to both improved performance and stronger self-efficacy beliefs (Bontempo and Napier, 2011; Chen et al., 2025; Wu and Liu, 2025). This proposed cascading mechanism, while theoretically coherent, was not empirically tested in the present study and warrants direct investigation in future research. As with the performance findings, however, the anxiety reduction cannot be attributed solely to the GenAI medium, because the individual practice format and unmonitored extracurricular exposure may have independently contributed to the lower anxiety levels observed in the EG. At the same time, the absence of a significant difference in learning engagement deserves attention. One plausible explanation is that peer role-play, despite its limitations in linguistic consistency, provides the social interaction and mutual accountability that sustain engagement. This interpretation resonates with Self-Determination Theory’s emphasis on relatedness—the basic psychological need for connection and belonging—as a key driver of intrinsic motivation (Deci and Ryan, 2000; Ryan and Deci, 2017). It suggests that the two practice modalities may support engagement through different psychological mechanisms—GenAI through autonomy and competence, peer interaction through relatedness—rather than producing fundamentally different levels of engagement.

The descriptive interaction analysis (RQ3) showed that students’ GenAI-mediated practice was structurally centered on meaning transfer and was primarily accompanied by clarification requests and self-repair. Because the ENA model aggregates co-occurrence patterns across the entire six-week period without week-by-week comparison, these findings characterize the overall structural profile of interaction rather than a developmental trajectory. The hub-and-spoke ENA network, with Close Rendition at its center and strong links to CCR, BR, and SER, suggests that students were not merely relaying information mechanically. Rather, they were actively negotiating meaning, monitoring comprehension, and adjusting their output in response to communicative difficulty—cognitive processes consistent with the metacognitive monitoring and self-regulation that characterize skilled interpreting performance (Gile, 2009). This pattern accords with Long's (1996) account of negotiation of meaning, as the linguistic complexity of the AI-generated input appears to have created authentic communicative pressure that elicited genuine interactional strategies. At the same time, the peripheral status of the human-AI collaboration codes, including PPA, HGH, and SAA, indicates that explicit metacognitive management of the AI system remained relatively limited during the intervention. This may reflect the novelty of the technology, students’ stronger orientation toward task completion than toward tool management, or the absence of explicit support for developing such metacognitive skills. It may also suggest that students need more structured guidance in how to direct, monitor, and recalibrate AI-based practice environments effectively. An apparent discrepancy exists between the very low frequencies of enacted human–AI collaboration codes in the interaction transcripts (PPA = 1, HGH = 4, SAA = 2) and the extensive agency behaviors reported in the interviews (RQ4). This discrepancy can be accounted for by the differing scopes of the two data sources. The coded transcripts captured only utterances produced during the interpreting task itself, where students were primarily occupied with meaning transfer; agency-related behaviors such as prompt adjustment and hallucination handling typically occurred between interpreting turns or at the beginning and end of practice sessions, moments that were either not transcribed or fell outside codeable interpreting exchanges. The interviews, by contrast, invited retrospective reflection across the full range of practice experiences, including pre-session setup, mid-session troubleshooting, and post-session review. The two data sources therefore provide complementary rather than contradictory evidence: the interaction analysis captures the structure of interpreting behavior during task performance, whereas the interviews capture the broader metacognitive and strategic repertoire that students drew upon across the entire practice cycle.

The qualitative findings (RQ4) complement and extend the quantitative results by offering a more nuanced account of the affordances, constraints, and adaptive behaviors associated with GenAI-mediated practice. Students’ concerns about role instability, the absence of nonverbal cues, and limited evaluative feedback echo issues identified in recent discussions of GenAI as an interactive practice partner (Goh and Aryadoust, 2025). Importantly, however, the data also show that students did not engage with the tool passively. Instead, they developed a range of agentic strategies, including prompt negotiation, self-directed difficulty scaffolding, and strategic repair of AI breakdowns. These behaviors align with Bandura's (2006) concept of psychological agency, which emphasizes individuals’ capacity to intentionally influence their own functioning and the conditions under which they operate. In the present study, agency appeared not only as an individual disposition but also as an emergent response to the opportunities and constraints of the technological environment—consistent with Bandura's (2006) view that agency is exercised within, and shaped by, the structural affordances and limitations of the environment in which action takes place.

### 5.2 Implications for practice

The findings of this study have several implications for the design of interpreting practice environments. First, GenAI-mediated practice should be conceptualized as a complement to, rather than a substitute for, traditional peer interaction, as the two modalities appear to engage different psychological mechanisms. GenAI can provide flexible, low-threat, and cognitively authentic practice opportunities that may support schema development and self-efficacy, whereas peer role-play may better satisfy the need for relatedness and social accountability. Moreover, the capacity to manage social pressure, respond to real-time criticism, and perform under interpersonal scrutiny is itself a core professional competence that interpreters must develop. The anxiety reduction afforded by GenAI, while beneficial in early training, should not be allowed to permanently shield learners from the evaluative demands of authentic professional settings. Healthy pressure from peers and instructors—including constructive criticism, spontaneous feedback, and the experience of being observed—can function as a productive driving force that builds resilience and emotional regulation skills (Bontempo and Napier, 2011). A well-designed curriculum should therefore use GenAI to build foundational confidence before progressively reintroducing the social-evaluative demands of face-to-face practice. Second, training programs should provide explicit instruction in prompt design, scenario specification, and AI management. The relatively limited presence of codes related to human-AI collaboration suggests that students may not automatically develop the metacognitive skills needed to use GenAI effectively. Targeted support may therefore be necessary if learners are to make strategic and autonomous use of such tools. Third, the substantial reduction in anxiety associated with AI-based practice suggests that GenAI may be especially valuable in the early stages of interpreter training, when learners are often most vulnerable to performance-related stress and when self-efficacy beliefs are still fragile (Bandura, 1997). In such contexts, GenAI may serve as a transitional practice partner that allows students to accumulate mastery experiences and build self-efficacy before engaging in more demanding face-to-face interaction. Finally, because GenAI does not yet provide consistently reliable evaluative feedback on interpreting quality, its value is likely to be maximized in blended designs that combine AI-mediated practice with instructor-guided debriefing, peer discussion, and structured reflective activities that help students evaluate their performance and consolidate learning.

In practical terms, these principles point to several concrete implementation strategies. Regarding curriculum integration, GenAI-mediated practice may be most effectively positioned as a supplementary out-of-class component rather than a replacement for in-class interaction: students complete AI-based dialogue sessions between classes and bring recordings or transcripts to instructor-led debriefing sessions. A progressive difficulty structure, moving from familiar, low-stakes scenarios (e.g., tourism) to specialized, high-pressure domains (e.g., legal or medical), allows learners to accumulate mastery experiences before confronting the cognitive demands of professional-level content. Regarding instructor preparation, educators need not become AI specialists, but they should receive targeted training in three areas: designing effective persona prompts that generate register-consistent and terminologically dense interlocutor discourse, identifying common AI malfunctions (role confusion, hallucination, unnatural pacing) so they can help students develop repair strategies, and integrating AI-generated transcripts into formative assessment activities. Regarding assessment, because GenAI does not yet provide reliable evaluative feedback on interpreting quality, assessment should remain instructor-driven. However, AI-generated practice transcripts offer a valuable data source: instructors can use them diagnostically to identify recurring error patterns, track the development of clarification and self-repair strategies over time, and provide targeted feedback grounded in students’ actual interaction data rather than in-class performance alone.

### 5.3 Ethical considerations in GenAI-mediated interpreting practice

Beyond the institutional ethical approval obtained for this study, the integration of GenAI into interpreting practice raises several ethical issues that warrant explicit discussion. The first concerns AI reliability and hallucination. As the qualitative findings revealed, the GenAI tool occasionally produced factually inconsistent information, switched languages unexpectedly, or departed from its assigned persona. In professional interpreting domains such as healthcare and legal services, where accuracy carries direct consequences for clients’ welfare, such hallucinations pose a non-trivial pedagogical risk: students may inadvertently rehearse responses to clinically or legally inaccurate information. Training programs should therefore ensure that students are explicitly taught to recognize AI-generated misinformation and that practice scenarios are not treated as sources of domain knowledge.

The second issue concerns data privacy. In this study, students used a commercial consumer-facing application on personal devices, and their voice recordings and interaction transcripts were processed by the platform. While the present study collected transcripts only with participants’ informed consent, the broader adoption of commercial GenAI tools in educational settings requires careful attention to institutional data governance policies, platform terms of service, and the protection of students’ biometric (voice) data. Institutions should evaluate whether commercial platforms meet their data protection requirements or whether API-based deployments with institutional data control are preferable.

The third issue concerns responsible educational use. The non-evaluative nature of GenAI interaction, identified by students as a key affordance, also carries a risk: without external performance feedback, students may develop false confidence in inaccurate renditions, a concern echoed in recent discussions of AI in interpreter training (Han et al., 2025; Wang and Wang, 2025). This reinforces the recommendation that GenAI-mediated practice should be embedded within instructor-supervised curricula rather than used as a standalone self-study tool, and that students should be guided to approach AI-generated interaction as practice, not as validation of their interpreting accuracy.

### 5.4 Limitations and future research directions

Several limitations should be acknowledged. First—and this constitutes the central limitation of the study—the two conditions were not matched on the amount of direct interpreting practice. Because CG students rotated among three roles every 15 min, each CG student spent only approximately 15 min per session in the interpreter role, whereas EG students interpreted continuously for the full 45-min practice period. EG students therefore accumulated roughly three times as much direct interpreting practice as CG students (approximately 270 versus 90 min across the six intervention weeks). Given that the amount of deliberate practice is among the most robust predictors of skill acquisition, the higher posttest performance of the EG may be attributable, in whole or in part, to this greater practice dosage rather than to the GenAI medium itself. Two further asymmetries compound this confound: the conditions also differed in practice format (individual versus triadic), and some EG students engaged in self-initiated practice outside class for which no exposure logs were collected. Because these factors co-vary with the instructional condition, the observed between-group differences—the performance findings in particular—cannot be attributed specifically to GenAI, and the possibility that they reflect practice volume rather than practice modality must be borne in mind throughout. Future research should equalize direct interpreting time across conditions (for example, by holding the interpreter role constant in peer role-play or by yoking the duration of AI-mediated practice to that of the peer condition) and should collect practice logs to quantify out-of-class exposure.

Second, the quasi-experimental design introduced multiple confounds that limit the interpretability of the between-group comparisons. Only one intact class was assigned to each condition, meaning that treatment effects are completely confounded with class-level characteristics such as group dynamics, peer composition, and classroom climate. The individual-level statistical tests (ANCOVAs) treat all 70 students as independent observations despite shared class membership, potentially violating the independence assumption and inflating Type I error rates. In addition, the two conditions were not matched on practice dosage (EG students interpreted continuously for 45 min; CG students interpreted for approximately 15 min due to role rotation), practice format (individual versus triadic), or extracurricular exposure (some EG students reported self-initiated practice outside class, but no exposure logs were collected). These confounds co-vary with the instructional condition; consequently, all between-group comparisons should be regarded as exploratory, and the observed differences cannot be attributed specifically to the GenAI medium. Future research should employ multi-class designs, equalize interpreting time, and monitor out-of-class practice to disentangle these factors.

Third, the eight-week intervention period may not have been long enough to capture longer-term developmental trajectories, particularly with respect to learning engagement and the gradual emergence of learner agency. Relatedly, the ENA analysis for RQ3 aggregated co-occurrence patterns across the entire six-week intervention period and did not include a week-by-week comparison; the findings therefore characterize the overall structural profile of students’ interaction strategies rather than a developmental trajectory. Future research should employ longitudinal ENA models (e.g., ordered network analysis; Tan et al., 2024) and delayed posttests to examine both temporal change in interaction patterns and the durability of the observed effects.

Fourth, the cognitive and motivational constructs invoked in the Discussion—including cognitive load, working-memory allocation, schema development, and self-efficacy—were not directly measured. All mechanistic explanations are therefore *post hoc* theoretical interpretations consistent with the observed data rather than empirically demonstrated pathways. Future research should incorporate dedicated measures of these constructs (e.g., dual-task paradigms for cognitive load, interpreting self-efficacy scales, transfer tasks for schema use) to test the proposed mechanisms directly.

Fifth, the sample size (*N* = 70, with 35 per group), while adequate for detecting medium-to-large effects, limits statistical power for detecting smaller differences and restricts the reliability of subgroup analyses.

Sixth, the study was conducted at a single institution with students from a relatively homogeneous linguistic background (Mandarin Chinese L1, English L2), and the GenAI tool used was optimized for Chinese-English interaction. Students at different proficiency levels, in different educational systems, or working with other language pairs—particularly less-resourced languages or those for which current LLMs perform less well—may yield different results. Multi-site research involving diverse language combinations, institutional contexts, and learner populations is needed to establish the external validity of these findings.

Seventh, the two conditions differed not only in the source of interlocutor input but also in modality: the control group practiced through face-to-face interaction with access to nonverbal cues, whereas the experimental group interacted with a voice-only AI system. This modality asymmetry means that the observed differences may partly reflect the absence of visual information rather than the AI-versus-human distinction alone. Future research should consider video-based GenAI interaction to reduce this asymmetry and isolate the effects of AI-generated content from those of interaction modality.

## Data Availability

The raw data supporting the conclusions of this article will be made available by the authors, without undue reservation.
