# Supplementary material for: GenAI-mediated immersive dialogue practice in liaison interpreting: a mixed-methods investigation of cognitive-affective outcomes and interactional processes
Source: Front Psychol. 2026 Sep 29;17:1914690. doi: 10.3389/fpsyg.2026.1914690 (PMC13658467; doi:10.3389/fpsyg.2026.1914690)
Supplement: Supplementary file 1 [file Supplementary_file_1.docx]

# Appendix A

**Bilingual Persona Prompt Template for GenAI-Mediated Immersive Dialogue Practice**

To ensure the GenAI (Doubao Voice Mode) accurately simulates a highly contextualized and unpredictable triadic liaison interpreting scenario, students were instructed to use the following parameterized prompt template before initiating the voice conversation.

1. The Master Prompt Template

"You are an advanced AI tasked with simulating a triadic liaison interpreting practice session. I am a student interpreter mediating the conversation. You must simultaneously act as two distinct interlocutors:

[Persona A - English Speaker]

- Role/Background: [Insert specific role, e.g., foreign client/patient/tourist]
- Language: English only
- Emotional State & Tone: [Insert tone, e.g., anxious, formal, demanding]

[Persona B - Chinese Speaker]

- Role/Background: [Insert specific role, e.g., local representative/doctor/police]
- Language: Mandarin Chinese only
- Emotional State & Tone: [Insert tone, e.g., professional, patient, defensive]

[Context/Topic] We are discussing: [Insert specific weekly theme and conflict/goal].

[Strict Rules of Engagement]

1. Alternate your personas. Only ONE persona speaks per turn.
2. CRITICAL: Wait for my interpretation after each turn. Do NOT translate your own speech into the other language.
3. React naturally to what the other persona says *based on my interpretation*.
4. To simulate a realistic environment, occasionally use idioms, domain-specific terms, or express hesitation/confusion if my interpretation is unclear.

If you understand, please start the role-play by having [Persona A or B] speak the first sentence."

2. Example Application (Week 5: Healthcare Consultation)

*(Example Input by an EG Student)* "You must simultaneously act as two distinct interlocutors: [Persona A - English Speaker] Role: An anxious Canadian patient experiencing severe abdominal pain. Language: English only. Tone: Worried, speaks relatively fast. [Persona B - Chinese Speaker] Role: An experienced Chinese gastroenterologist. Language: Mandarin Chinese only. Tone: Professional, calm, uses medical terms. [Context] We are in a hospital. The patient is describing symptoms, and the doctor is diagnosing suspected acute appendicitis. [Strict Rules...] *(same as above)* Please start the role-play by having the Canadian patient speak the first sentence."

# Appendix B

Table B1. *Analytic Rubric for Liaison Interpreting Performance Assessment*

| **Band** | **Score Range** | **Performance Descriptor** |
| --- | --- | --- |
| ***Dimension 1: Informational Accuracy and Completeness*** *(50 points)* | | |
| *Assesses the degree to which the interpreter faithfully transfers the propositional content of each source utterance, preserving key information without unjustified omissions, additions, or distortions.* | | |
| Excellent | 43–50 | All essential propositions are transferred accurately and completely. No meaning-altering omissions, additions, or distortions. Minor details (e.g., hedging markers, discourse fillers) may be absent without affecting the communicative outcome. |
| Good | 35–42 | Most essential propositions are transferred accurately. Occasional minor omissions or imprecisions are present but do not alter the overall message or mislead either interlocutor. Key figures, names, and terms are rendered correctly. |
| Adequate | 27–34 | The core message is generally conveyed, but noticeable omissions, simplifications, or inaccuracies occur in several turns. Some information loss is evident, though the general direction of the exchange remains recoverable. |
| Inadequate | 18–26 | Frequent omissions and distortions substantially compromise the transfer of meaning. Important propositions are missed or altered, leading to potential misunderstanding between interlocutors. |
| Poor | 0–17 | The rendition bears little resemblance to the source. Pervasive omissions, fabrications, or misrepresentations render the interpretation unreliable for communicative purposes. |
| ***Dimension 2: Target Language Quality and Delivery*** *(20 points)* | | |
| *Evaluates the linguistic accuracy, fluency, and delivery of the interpreter’s output in both target languages (Chinese and English), including grammar, lexical appropriateness, pronunciation/prosody, and pacing.* | | |
| Excellent | 17–20 | Output is grammatically accurate, lexically appropriate, and delivered fluently in both languages. Pronunciation and prosody are natural. Pacing is well calibrated to the interlocutors’ needs. Register is consistently appropriate. |
| Good | 13–16 | Output is largely accurate and fluent, with occasional grammatical slips, lexical approximations, or minor hesitations that do not impede comprehensibility. Register is generally appropriate. |
| Adequate | 9–12 | Output is comprehensible but marked by recurrent grammatical errors, limited lexical range, noticeable hesitations, or inconsistent register. Delivery occasionally impedes the flow of the interaction. |
| Inadequate | 5–8 | Output contains frequent grammatical errors and lexical gaps that compromise comprehensibility. Delivery is noticeably disfluent, with prolonged pauses or false starts that disrupt the communicative exchange. |
| Poor | 0–4 | Output is largely incomprehensible due to pervasive errors in grammar, vocabulary, and pronunciation. Delivery is halting or incoherent. |
| ***Dimension 3: Interactional Management*** *(30 points)* | | |
| *Examines the interpreter’s ability to coordinate the triadic exchange, including turn-taking management, the use of clarification and repair strategies, and appropriate responses to pragmatic shifts such as changes in topic, register, or interlocutor affect (Wadensjö, 2014).* | | |
| Excellent | 26–30 | The interpreter manages the triadic interaction seamlessly. Turns are allocated clearly and efficiently. Clarification requests and repairs are deployed proactively and appropriately when comprehension is at risk. The interpreter responds flexibly to pragmatic shifts (e.g., topic change, emotional escalation, register shift) and actively facilitates mutual understanding between interlocutors. |
| Good | 21–25 | The interpreter manages most turns effectively and maintains the flow of the exchange. Clarification strategies are used when needed, though occasionally with slight delay. Pragmatic shifts are generally recognized and handled, with minor lapses in coordination. |
| Adequate | 15–20 | The interpreter maintains a basic triadic structure but shows inconsistency in turn management. Clarification strategies are underused or applied after a noticeable delay. Some pragmatic shifts are missed or responded to rigidly, resulting in occasional communicative friction. |
| Inadequate | 9–14 | Turn management is disorganized, with frequent overlapping, long silences, or failure to signal turn allocation. Clarification strategies are rarely used or ineffective. The interpreter struggles to adapt to pragmatic changes, resulting in breakdowns in the communicative flow. |
| Poor | 0–8 | The interpreter fails to maintain the triadic structure. Interlocutors are left without clear turn allocation. No observable use of clarification or repair strategies. The interaction effectively collapses into disconnected segments. |

*Note.* Adapted from Hale (2007), Pöchhacker (2022), and Wadensjö (2014). Total score = 100. Raters assign a holistic score within each band for each dimension based on the overall match between the performance and the descriptor. The three dimensions are weighted as follows: Informational Accuracy and Completeness = 50%, Target Language Quality and Delivery = 20%, and Interactional Management = 30%.
